# A Biomechanical Comparison of Expansive Pedicle Screws for Severe Osteoporosis: The Effects of Screw Design and Cement Augmentation

**DOI:** 10.1371/journal.pone.0146294

**Published:** 2015-12-31

**Authors:** Ching-Lung Tai, Tsung-Ting Tsai, Po-Liang Lai, Yi-Lu Chen, Mu-Yi Liu, Lih-Huei Chen

**Affiliations:** 1 Graduate Institute of Medical Mechatronics, Department of Mechanical Engineering, Chang Gung University, Kweishan, Taoyuan, Taiwan; 2 Department of Orthopedic Surgery, Bone and Joint Research Center, Chang Gung Memorial Hospital, Chang Gung University College of Medicine, Kweishan, Taoyuan, Taiwan; Van Andel Institute, UNITED STATES

## Abstract

Expansive pedicle screws significantly improve fixation strength in osteoporotic spines. However, the previous literature does not adequately address the effects of the number of lengthwise slits and the extent of screw expansion on the strength of the bone/screw interface when expansive screws are used with or without cement augmentation. Herein, four designs for expansive pedicle screws with different numbers of lengthwise slits and different screw expansion levels were evaluated. Synthetic bones simulating severe osteoporosis were used to provide a comparative platform for each screw design. The prepared specimens were then tested for axial pullout failure. Regardless of screw design, screws with cement augmentation demonstrated significantly higher pullout strength than pedicle screws without cement augmentation (p < 0.001). For screws without cement augmentation, solid screws exhibited the lowest pullout strength compared to the four expansive groups (p < 0.01). No significant differences in pullout strength were observed between the expansive screws with different designs (p > 0.05). Taken together, our results show that pedicle screws combined with cement augmentation may greatly increase screw fixation regardless of screws with or without expansion. An increase in both the number of slits and the extent of screw expansion had little impact on the screw-anchoring strength. Cement augmentation is the most influential factor for improving screw pullout strength.

## Introduction

Transpedicular screws have been widely used to achieve stabilization of the posterior spine for treatment of a variety of spinal disorders [1–5]. However, the loosening or failure of fixation screws resulting from inadequate screw holding power or excess load of the repaired vertebrae is not uncommon, particularly in patients with osteoporosis [6–8]. Loosening or failure of the screws might induce sagittal collapse of the spinal column or painful kyphosis [9,10].

Numerous reports have demonstrated that several factors, such as bone mineral density, screw design, cement augmentation, and surgical technique, are related to screw pullout resistance [11–18]. Among the numerous variables that affect screw fixation strength, bone mineral density plays the largest role [19,20]. Consequently, for patients with severe osteoporosis, ensuring high pullout strength for screws in non-augmented osteoporotic bone is a challenge for surgeons. Extensive work to increase screw fixation strength in poor-quality bone has been performed. Polymethylmethacrylate (PMMA) bone cement is the most readily available and cost effective material for augmentation and has been used in many clinical orthopedic applications [21–23]. To date, attempts at segmental stabilization and fusion have focused primarily on surgical techniques for screws augmented with PMMA [12,13,21,24], calcium phosphate [25,26] and calcium sulfate [27,28] in osteoporotic bone.

When PMMA is applied to spinal vertebrae with osteoporosis, solid screws with retrograde cement pre-filling are generally used to achieve robust screw fixation in osteoporotic bone. In such augmentation techniques, PMMA is squeezed directly into the prepared pilot hole in the vertebral body prior to screw insertion. The solid pedicle screw is then inserted into the cement to enhance screw-anchoring strength [12]. Another insertion technique involves the use of a perforated screw with PMMA augmentation, allowing the injection of cement through the perforation to enhance screw fixation strength [13,24,29]. Recently, efforts have focused on using expansive screws to enhance postoperative stability. The expansive pedicle screw was developed primarily to improve fixation in bone that has been compromised by osteoporosis or pedicle screw revision [30–32]. The expansive screw has a lengthwise hollow in which an expansion pin can be inserted. The screw tip is divided lengthwise to form separated flanges, which allows for flange expansion at the screw tip after pin insertion, increasing screw-anchoring power. In practice, the expanded screw can compress bone at the screw/bone interface with the anterior screw tip, which is believed to provide more screw thread engagement with the vertebral bone than a conventional pedicle screw of identical size.

Although extensive studies have demonstrated improvement in pullout strength using expansive screws, reports that address the influences of design parameters such as the degree of screw expansion and the number of lengthwise wings on the axial pullout strength of the expansive screws, with or without cement augmentation, are lacking. In this study, we address three features that might alter the pullout strength of expansive screws: 1) the effect of expansive range, 2) the effect of the number of the lengthwise wings and 3) the effect of insertion techniques with or without cement augmentation.

## Materials and Methods

### Synthetic bone samples

Synthetic composite bone (test block, model #1522–507, Pacific Research Laboratory Inc., Vashon Island, WA, USA) was chosen as the test object to represent human vertebrae with severe osteoporosity, which minimized experimental variation caused by the variability of bone properties and morphometry. The synthetic bone was constructed from open-cell rigid polyurethane foam with a density of 0.09 g/cm^3^. These test blocks provided a homogeneous and consistent material similar to human cancellous bone with extreme osteoporosis [33]. The test blocks used in the pullout tests had dimensions of 8 cm x 6 cm x 4 cm. For all of the specimens, a pilot hole was drilled into the test block using a 3-mm drill bit prior to screw insertion.

### Bone screws

**Fig 1:**
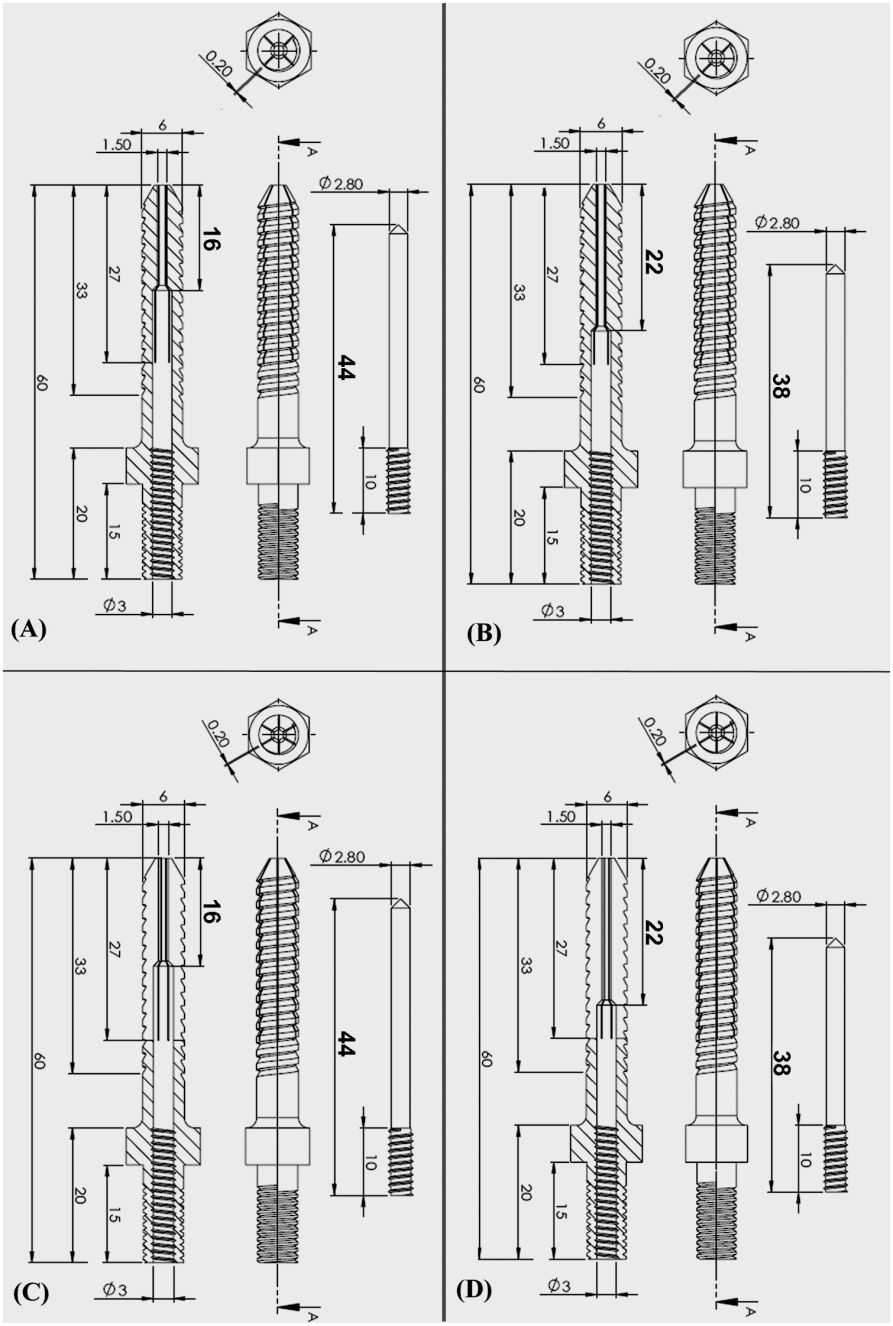
Schematic drawings showing four types of expandable screws: (A) 4-slit with 16-mm EEL, (B) 4-slit with 22-mm EEL, (C) 6-slit with 16-mm EEL, and (D) 6-slit with 22-mm EEL. The EEL was defined as the length from the point of diameter change of the internal hole to the screw tip. All of the screws had the same outer diameter of 6 mm, a length of 40 mm from hub to tip, a thread pitch of 2 mm and a thread depth of 0.8 mm. The lengthwise slit length from the screw tip was 27 mm for all screws. All of the expansive screws had an internal hole 3 mm from the screw head, which was connected to a smaller hole (1.5 mm in diameter) that extended to the screw tip.

**Fig 2:**
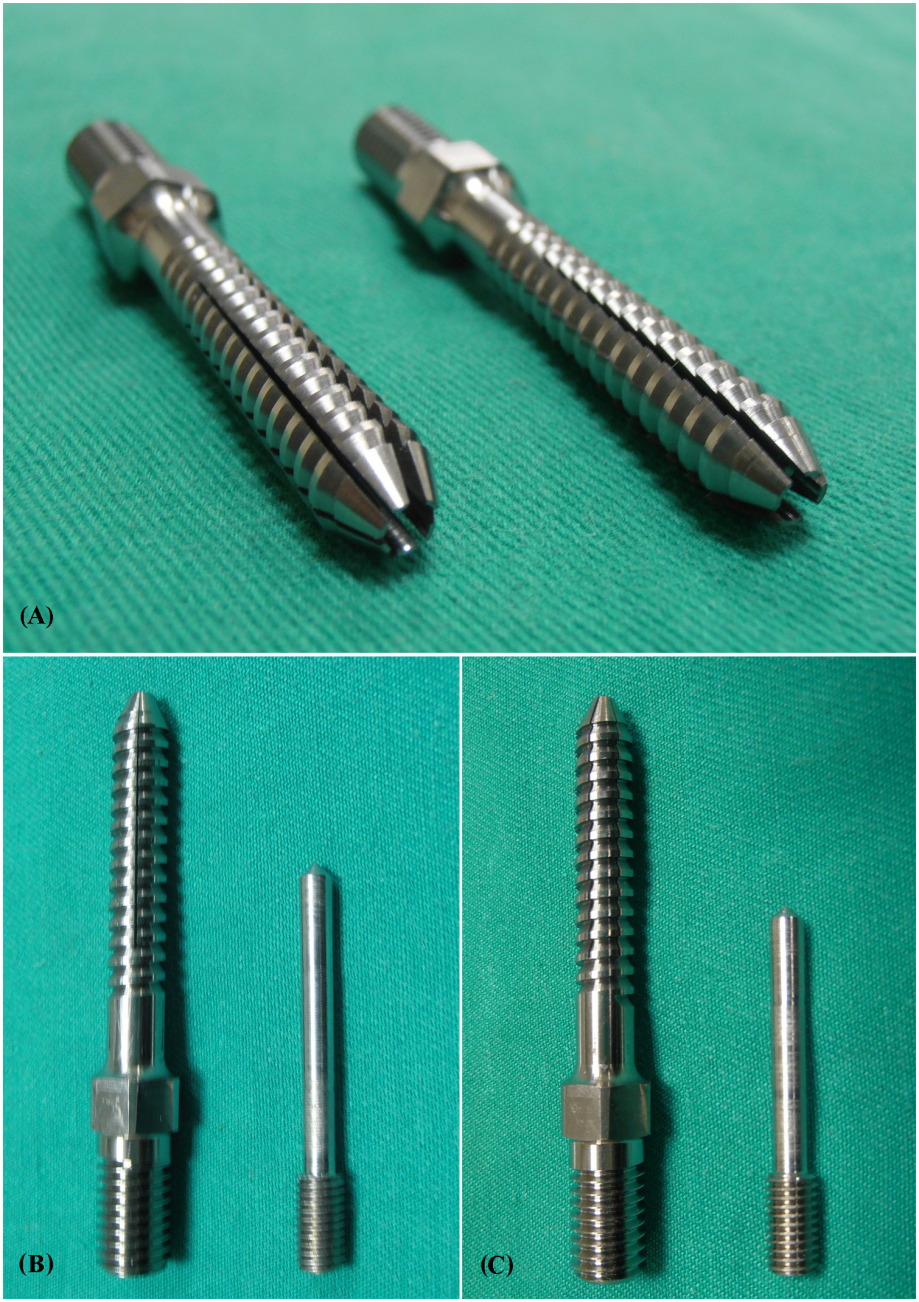
Photographs that show expansive screws with 6 (left) and 4 (right) separated lengthwise slits (A), and expansive screws with different EELs: (B) 16-mm EEL (with an inner pin of 44-mm in length) and (C) 22-mm EEL (with an inner pin of 38-mm in length). The insertion pin was inserted into the interior of the screw to open the fins at the tip of the expansive screws. The difference in EEL affects the range of screw expansion following pin insertion.

Five screw designs were employed in the present study: solid screws (control) and four types of expansive screws. All of the screws had the same outer diameter of 6 mm, a length of 40 mm from hub to tip, a thread pitch of 2 mm and a thread depth of 0.8 mm. The lengthwise slit length from the screw tip was 27 mm for all of the screws. All of the expansive screws had an internal hole 3 mm from the screw head, which was connected to a smaller hole (1.5 mm in diameter) that extended to the screw tip (Fig 1). Four types of expansive screws were studied: 4-slit or 6-slit with 16-mm or 22-mm EELs. The EEL was defined as the length from the point of diameter change of the internal hole to the screw tip (Fig 1). The 4-slit and 6-slit screws had 4 or 6 lengthwise slits, respectively, which started from the screw tip to form four or six anterior fins (Fig 2A). For screws with 16-mm or 22-mm EELs, the length from the point of diameter change of the internal hole to the screw tip was 16 mm or 22 mm, respectively. The anterior portion of the screw was split lengthwise by an inner pin to form four or six separated anterior fins. The difference in EEL affects the range of screw expansion following pin insertion. The inner pins were all 3 mm in diameter, and they were 44 mm or 38 mm in length for screws with 16 mm or 22 mm EELs, respectively (Fig 2B and 2C). The insertion pin was inserted into the interior of the screw to open the fins at the tip of the expansive screws. Expansive screws with various design parameters are illustrated in (Fig 1).

### Allocation of the specimens

The allocation of specimens to experimental groups is presented in Table 1. The following ten combinations of screw design and augmentation technique were tested (six replicates in each group):

Group 1: Solid screws, cementless.
Group 2: Expansive screws, 4-slit with 16 mm EEL, cementless.
Group 3: Expansive screws, 4-slit with 22 mm EEL, cementless.
Group 4: Expansive screws, 6-slit with 16 mm EEL, cementless.
Group 5: Expansive screws, 6-slit with 22 mm EEL, cementless.
Group 6: Solid screws, cemented.
Group 7: Expansive screws, 4-slit with 16 mm EEL, cemented.
Group 8: Expansive screws, 4-slit with 22 mm EEL, cemented.
Group 9: Expansive screws, 6-slit with 16 mm EEL, cemented.
Group 10: Expansive screws, 6-slit with 22 mm EEL, cemented.

**Table 1:** Allocation of the specimens to experimental groups.

Group	Screw Type	Slit Number	EEL (mm)	Screw Expansion	Augmentation	Specimen Number
1	Solid	NA	NA	NA	None	6
2	Expandable	4	16	Yes	None	6
3	Expandable	4	22	Yes	None	6
4	Expandable	6	16	Yes	None	6
5	Expandable	6	22	Yes	None	6
6	Solid	NA	NA	NA	PMMA augmentation	6
7	Expandable	4	16	Yes	PMMA augmentation	6
8	Expandable	4	22	Yes	PMMA augmentation	6
9	Expandable	6	16	Yes	PMMA augmentation	6
10	Expandable	6	22	Yes	PMMA augmentation	6

### Specimen Preparation

**Fig 3:**
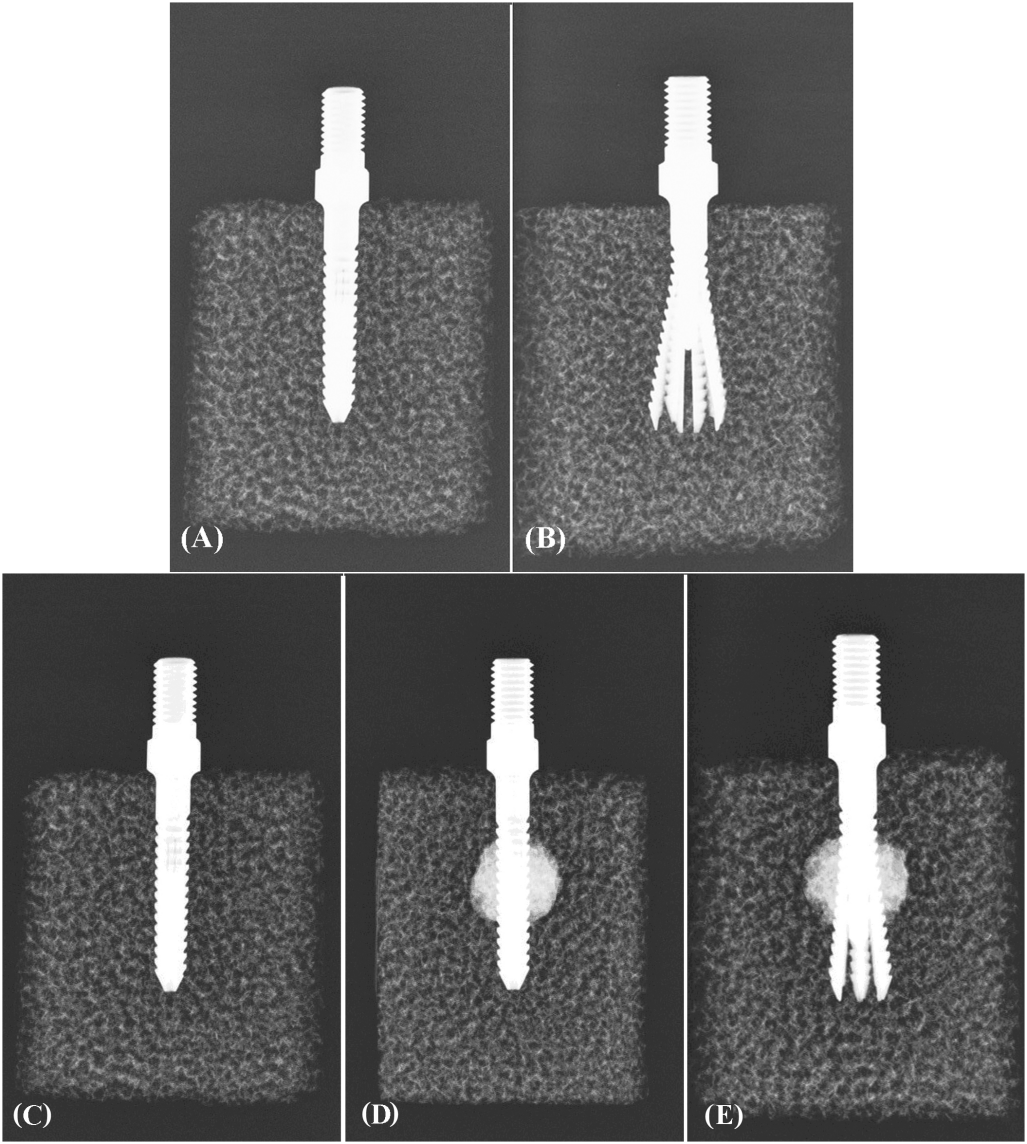
Radiological photograph showing the procedure for an expansive screw with different preparations. For screws without cement augmentation (top): (A) Insertion of the expansive screw into the test block. (B) Insertion of inner pin to achieve screw expansion. For screws with cement augmentation (bottom): (C) insertion of the expansive screw into the test block. (D) Cement injection after screw insertion (prior to expansion). (E) Insertion of the inner pin to achieve screw expansion.

Prior to screw insertion, a pilot hole was drilled into the test block using a 3-mm drill bit, and a pedicle screw was then inserted into the test block through the prepared pilot hole. The insertion rate for all of the screws was 3 rev/min [34], and a countdown timer was used to measure the screw insertion rate. All screws were inserted to the same depth using a consistent depth gauge, and radiological examinations were performed to check the implanted screw depths. For screws without cement augmentation, an inner pin was inserted into the central hole of the screw to achieve screw expansion. The radiological examination of expansive screws without cement augmentation is shown in Fig 3 (top). For screws with cement augmentation, Osteobond bone cement (Zimmer, Warsaw, IN) was mixed at room temperature and introduced into the expansive screws using a self-designed cement injector system that exerted pressure on the cement. The cement injector consisted of a cement gun, syringe, adapter and expansive screw. One minute after the cement powder and monomer were mixed, the liquid-phase cement was transferred into a 10-ml syringe, which was then inserted into the cement gun. An adapter was used to connect the syringe to the expansive screw. For all specimens, a total of 3 ml of cement was injected into the expansive screw. Following cement injection, the inner pin was inserted into the central hole of the screw to achieve screw expansion. The radiological examination of the expansive screws with cement augmentation is shown in Fig 3 (bottom). For all of the expansive screws after expansion, the diameter at the screw tip was measured using a digital micrometer with range of 0 to 25 mm and a resolution of 0.001 mm (Model PB-1B, Mitutoyo, Tokyo, Japan).

### Biomechanical tests

Screw pullout tests were conducted according to the ASTM F543-07 testing standards [34]. Each prepared specimen was tested for failure in axial pullout using an Instron testing machine (model 5544, Instron Inc., Canton, MA, USA). The test block, with a screw inserted, was placed on a specially designed universal fixture with a self-aligning function to ensure vertical pullout alignment. The heads of the screws were fixed in a 10 mm diameter cylindrical rod with an inner thread that matched the outer thread of the screw head. The cylindrical rod was then clamped to the testing machine. After the specimens were mounted, a pullout force was applied at a constant crosshead rate of 5 mm/min [34]. The force acting on the screw during testing was continuously recorded in 0.1 mm increments until the peak pullout resistance was reached. The peak force recorded during the pullout test was defined as the maximum pullout load sustained before failure. Six trials for each screw fixation configuration were performed, and the mean value of the maximum pullout load of the six trials was determined.

### Statistical analysis

All of the measurements were collected in six trials and are expressed as the mean ± standard deviation (SD). To evaluate the effects of screw design (4-slit compared to 6-slit, 16-mm EEL compared to 22-mm EEL) and different modes of screw implantation (cemented compared to cementless) on the stability of spinal fixation, the magnitudes of the ultimate pullout force were statistically compared. ANOVA test with post-hoc analyses were performed to evaluate difference among groups. Differences were considered significant at p < 0.05.

## Results

**Fig 4:**
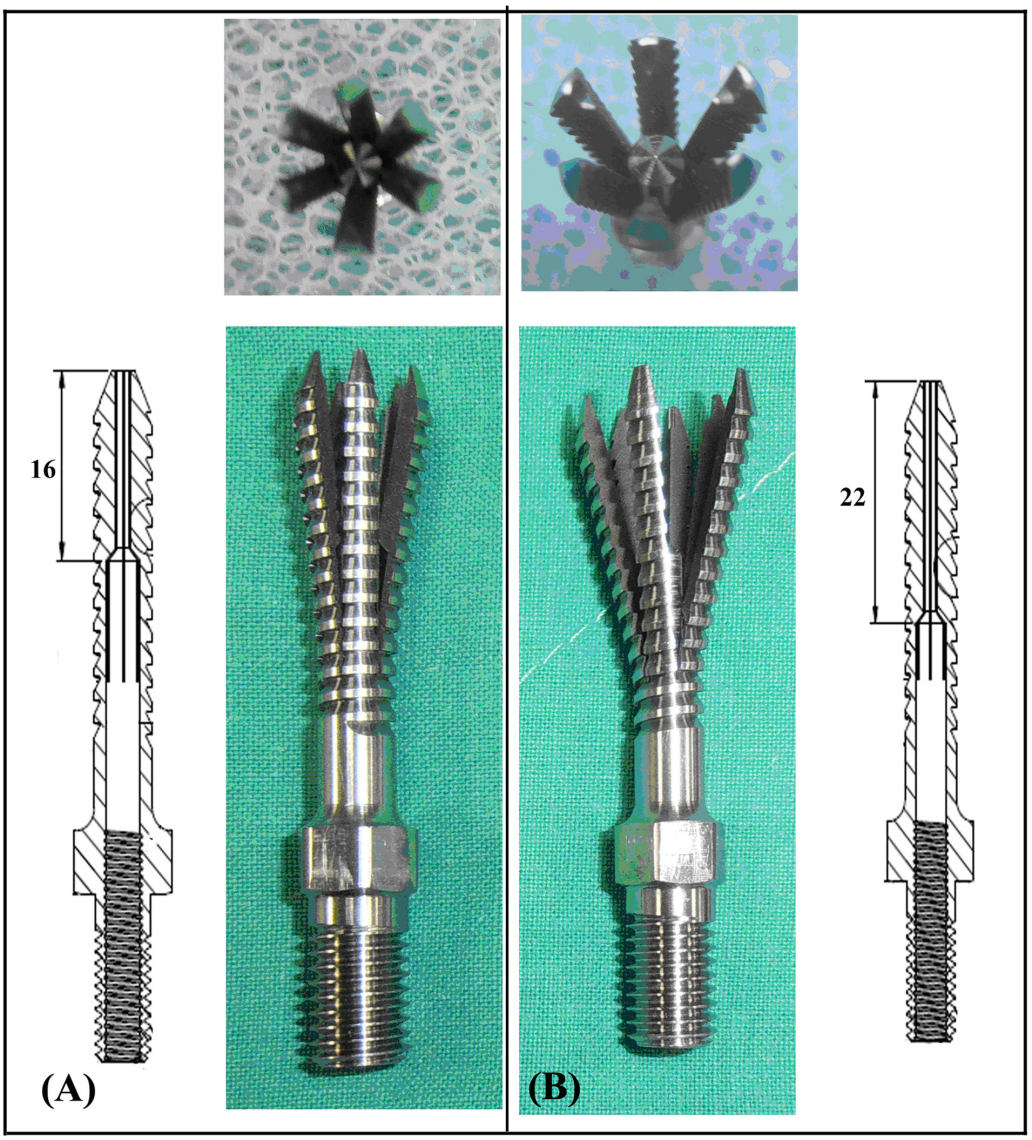
Photographs showing the pedicle screws after expansion: (A) 6-slit screw with 16-mm EEL and (B) 6-slit screw with 22-mm EEL. A pedicle screw with a 22-mm EEL has a larger expansion range (top).

**Fig 5:**
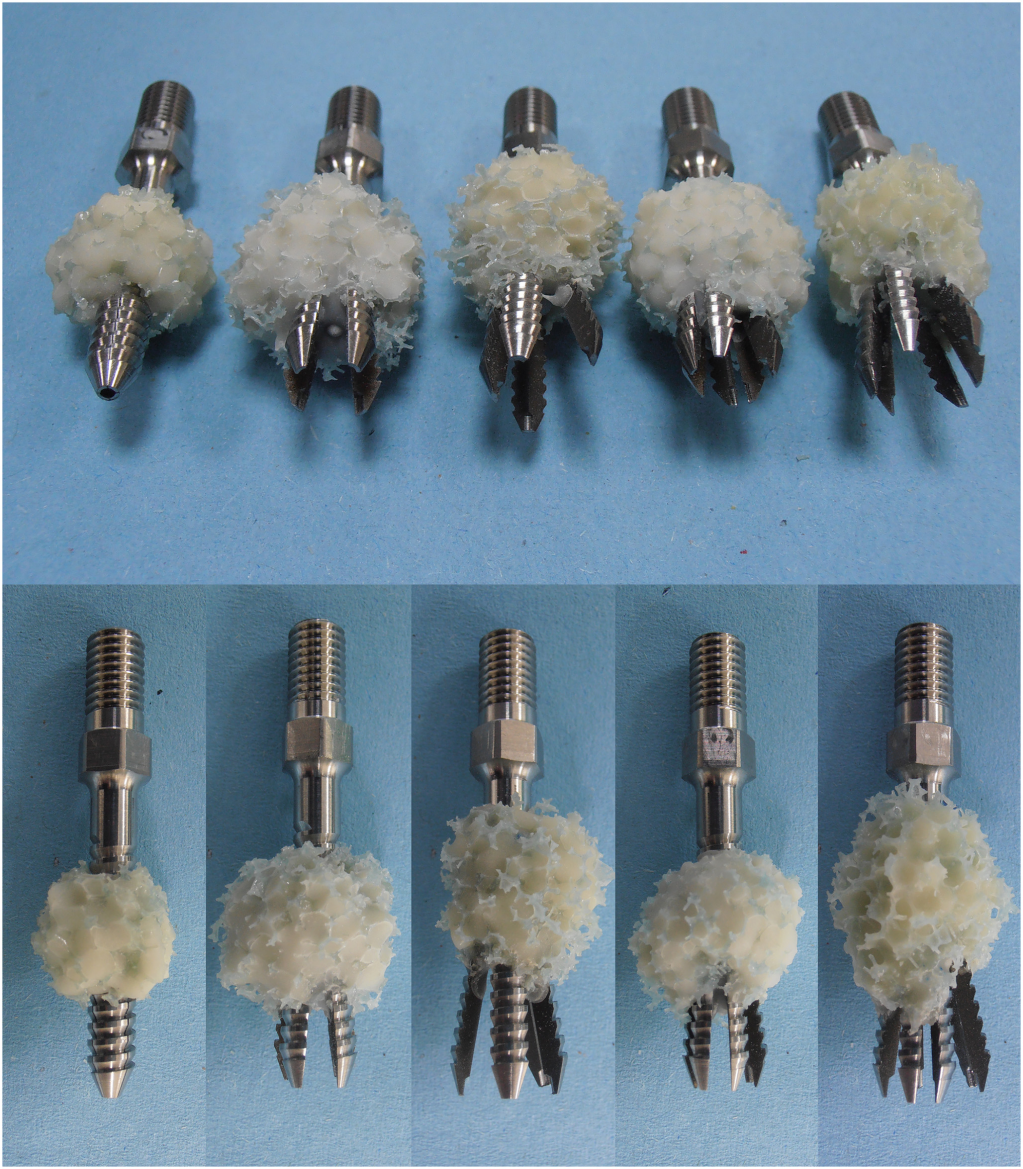
Photographs showing various cemented screws after the pullout tests. Left to right: solid, 4-slit with 16-mm EEL, 4-slit with 22-mm EEL, 6-slit with 16-mm EEL, and 6-slit with 22-mm EEL. A larger expansion range was observed for screws with 22-mm EELs compared with screws with 16-mm EELs and that cement infiltration into the open cell of the test block led to the formation of a cement/bone composite structure. Regardless of the number of slits (4 or 6), the cement/bone composite structure was distributed closer to the screw head for screws with a larger expansion range (22-mm EELs).

**Fig 6:**
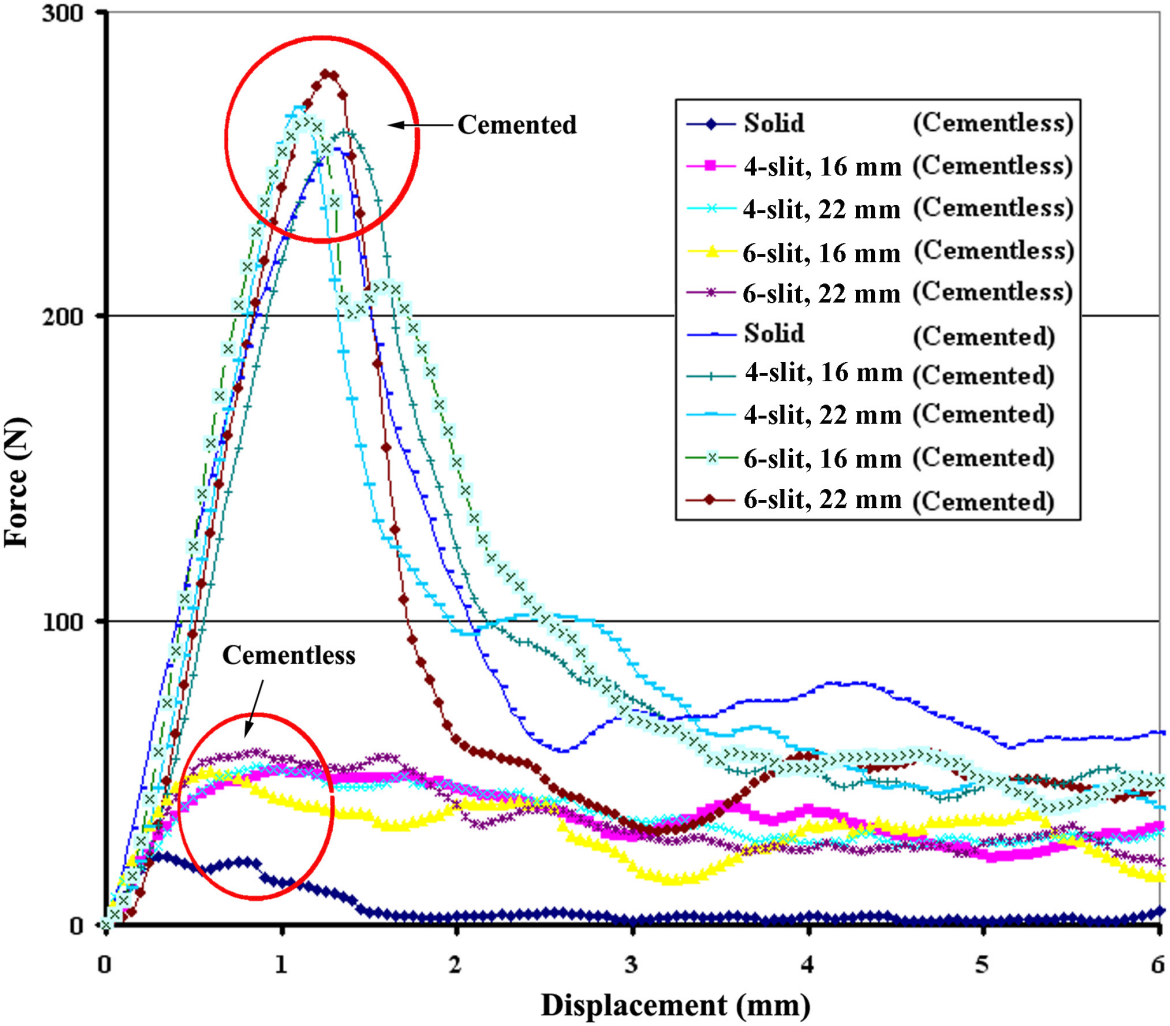
Typical force-displacement curve for various types of expansive screws with or without cement augmentation.

**Fig 7:**
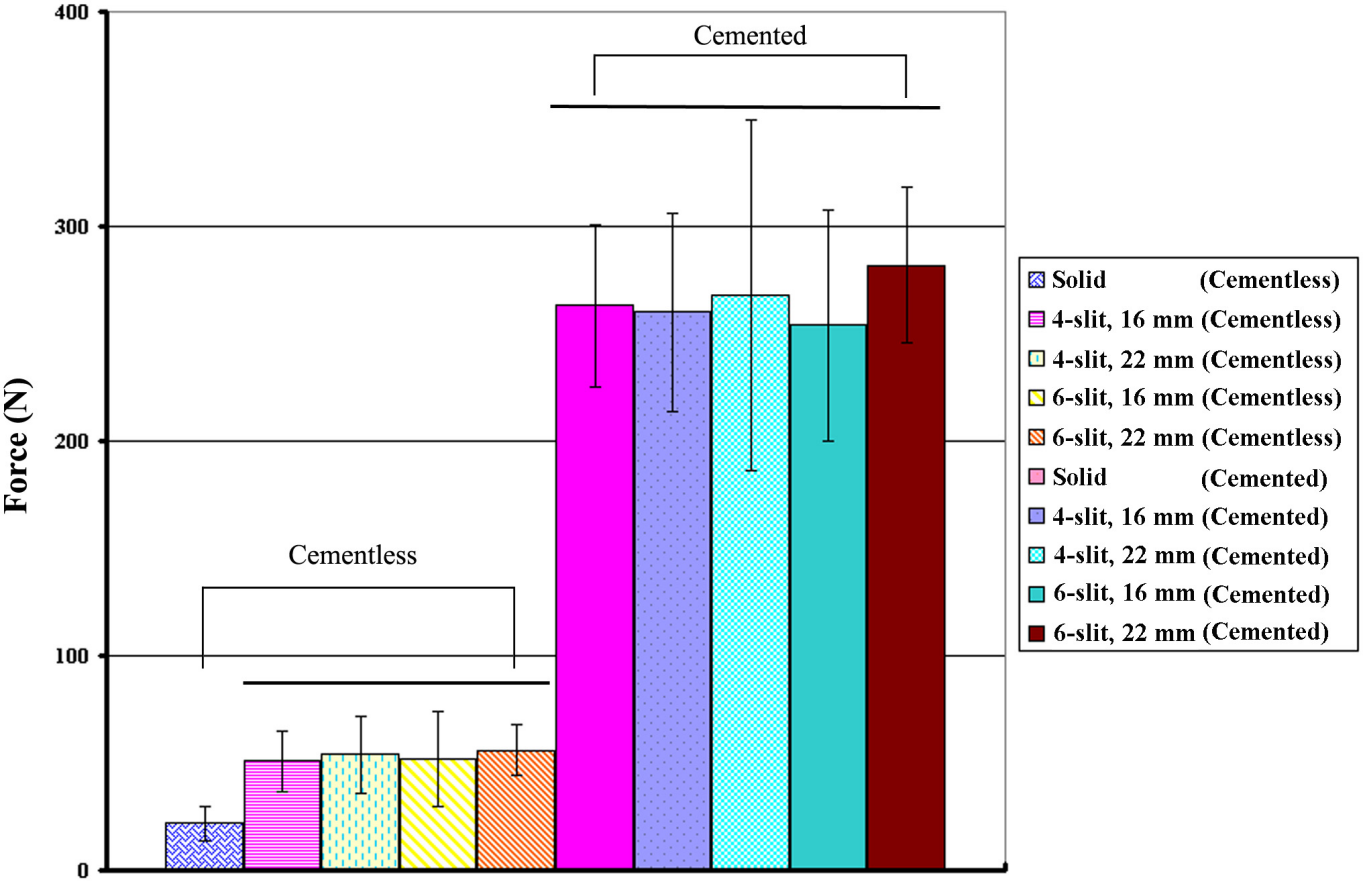
The mean ultimate pullout strengths of various types of expansive screws.The vertical lines represent the standard deviations. Horizontal lines above the bars indicate groups that were not significantly different from each other. Regardless of the screw design (screws with 4 or 6 slits and EELs of 16 or 22 mm, or a solid screw), screws with cement augmentation demonstrated significantly higher pullout strengths than did pedicle screws without cement augmentation (p < 0.001).

The average diameters at the screw tip after screw expansion for 4-slit screws with 16- and 22-mm effective expansion lengths (EELs) were 10.61 ± 0.54 mm and 13.02 ± 0.46 mm (p < 0.001), respectively. For 6-slit screws with 16- and 22-mm EELs, the average diameters were 11.07 ± 0.67 mm and 13.24 ± 0.28 mm (p < 0.001), respectively. Expansive screws with 16- and 22-mm EELs after expansion are shown in Fig 4. The results revealed that screws with longer EELs achieved larger expansion ranges, whereas no significant difference in expansion range was observed between 4-slit and 6-slit designs with the same EEL (p > 0.05). The physical examination of various screws with cement augmentation after pullout tests is shown in Fig 5. Observations of the failed specimens after pullout tests also revealed that a larger expansion range was observed for screws with 22-mm EELs compared to screws with 16-mm EELs. Furthermore, cement infiltration into the open cell of the test block led to the formation of a cement/bone composite structure. Regardless of the number of slits (4 or 6), the cement/bone composite structure was distributed closer to the screw head for screws with a larger expansion range (22-mm EELs). A typical force-displacement curve and the mean maximum pullout strength for various screws with and without cement augmentation are shown in Figs 6 and 7, respectively. Regardless of the screw design (4 or 6 slits and EELs of 16 or 22 mm, or a solid screw), screws with cement augmentation exhibited significantly higher pullout strengths than did pedicle screws without cement augmentation (p < 0.001). However, no significant difference in pullout strength was observed among the five cemented groups (p > 0.05). Additionally, in the cementless group, solid screws showed the lowest pullout strength compared to the four expansive groups (p < 0.01), whereas no significant difference was observed among the four expansive groups (p > 0.05). Furthermore, for a given screw fixation technique (cemented or cementless), no significant difference in pullout strength was observed among expansive screws with different numbers of slits or degrees of screw expansion (p > 0.05). The difference in both the number of slits and expansive extent had little impact on screw-anchoring strength. Cement augmentation was the most influential factor improving screw pullout strength.

## Discussion

Pedicle screw fixation remains a challenge for patients with severely osteoporotic spines. The screw-anchoring strength is greatly compromised in poor-quality bone, which can result in subsequent screw loosening or fixation failure. Numerous reports have demonstrated that expansive pedicle screws can significantly enhance the initial fixation strength compared to conventional screws [30,31,35]. Our results are consistent with those of Cook *et al*. [30], who reported that PMMA injection through an expanded screw increased pullout strength in severely osteoporotic bone compared to a non-cemented expandable screw. They concluded that PMMA made notable contributions to screw stability in osteoporotic bone. In this study, we further demonstrated that although an expansive screw could markedly enhance screw stability in the absence of cement, the increase in pullout strength was less than that of a traditional screw with cement augmentation unless the expansive screw was used with cement augmentation.

Although previous literature has demonstrated that expansive screws effectively enhance screw-fixation strength, our results demonstrate that expansive screws combined with cement augmentation may further increase screw-fixation strength compared to an expandable screw alone. Increasing both the number of slits and the extent of screw expansion had little impact on screw-anchoring strength. For expansive screws, we did not observe any noticeable difference in pullout strength among the various screw designs (4-slit or 6-slit, 16- or 22-mm EEL) for screws using the same augmentation technique (cemented or cementless). However, regardless of the screw design, pullout strength in the cemented groups was significantly greater than in the cementless groups (Fig 7). This study demonstrates that cement augmentation has a greater contribution to the initial stability of pedicle screws than the numerous design factors of expansive screws.

Observations of the failed specimens after the pullout tests indicated that cement infiltration into the open cell of the test block led to the formation of a cement/bone composite structure (Fig 5). All of the failures occurred at the composite/bone interface, but the composite remained well bound to the screws. This phenomenon can be attributed to the much higher screw/composite interfacial strength compared with the composite/bone interfacial strength. During screw pullout, the screw-bonded composite provided enormous pullout resistance due to interference between the cement/bone composite and the surrounding bone. For expansive screws, however, the mechanism underlying the increase in pullout strength can be attributed to radial compression toward the expanding direction that was created when the separated fins expanded, creating an increase in the bone mineral density around the fins. The separated fins provided a radial compression force on the surrounding bone and thus increased the pullout strength. In this study, we observed that regardless of screw type, there was a significant increase in the pullout strength of cemented screws relative to expansive screws without PMMA augmentation using artificial osteoporotic bones (Fig 7). This result implies that the screw-bonded composite is more effective at increasing the pullout strength of cemented screws than the radial compression caused by expansive screws. Our results demonstrate that cement extrusion into a test block to form a cement/bone composite structure should be the dominant factor determining pullout strength, rather than the design of screws with an expansive function. However, although cement augmentation increases the screw-anchoring power, observation of the specimens after the pullout tests indicated that the extruded cement had a more proximal distribution for screws with a larger extent of expansion (Fig 5). This result implies that the use of expansive screws with larger extents of expansion has an associated risk of cement leakage into the spinal canal. Expansive screws with smaller final expansion sizes might be beneficial for reducing the risk of cement leakage.

In this study, synthetic osteoporotic bones (test blocks) were used as a substitute for human osteoporotic vertebrae. Because variations in the apparent density, trabeculae orientation and mechanical properties of cancellous bone within and among specimens are large, numerous tests are required to isolate the effects of screw design when cancellous bone is used. The use of synthetic cancellous bone simplified the experimental setup, thus limiting experimental error. Cancellous bone is generally reported to have a density in the range of 0.09 to 1.25 g/cm^3^ [33]. The test blocks used in this study had a density of 0.09 g/cm^3^ and were chosen to model human osteoporotic vertebrae. Although the mechanical tests did not measure the pullout strength from actual cadaveric vertebrae, they measured the screw/cement/bone interfacial strength, which allowed examination of the relative differences between different screw designs. Unlike a more clinically relevant loading combining pullout, transeverse, moment loading performed by Choma et al, who investigated bioactive cements augmenting pedicle screw resistance to loosening using multicomponent loading [36], this study used axial pullout strength of pedicle screws to evaluate the screw-anchoring power when expansive screws were applied with or without cement augmentation. Although axial pullout of pedicle screws is not the only mechanism responsible for clinical failure, the pullout strength of pedicle screws has been extensively studied using cadaver bone [10,30,31], animal bone [28,32], and artificial materials [12,13,26]. To date, it is a popular experimental method and has been widely accepted for evaluation of screw/bone interface strength of pedicle screws in different screw designs. This method provides a standard platform to check the uniformity of the screws and to compare the pullout strength of different screw designs. Nevertheless, the pullout strength of pedicle screws might be affected by screw design, bone mineral density, bone structures, inserted condition, and the preparation of pilot holes. For a fair comparison among different screws, adequate control of these variables should be done with caution.

Our study has some limitations. First, the pedicle screws were manufactured in-house using only one type of metal, diameter, thread and outer geometry (cylindrical). The possible effects of variations in the above-mentioned properties were not considered. Second, a synthetic composite bone was used as a substitute for human bone. Although synthetic bone provides a platform for the comparison of screw fixation stability, there must be some differences between the mechanical characteristics of the synthetic bone and those of actual bone; therefore, extrapolation of our results to clinical applications should be performed with caution. Third, only open-cell rigid polyurethane foam with a density of 0.09 g/cm^3^ was used to simulate a cadaveric spinal bone with extreme osteoporosis. Bone density might significantly influence differences in the screw/bone interfacial strength among various screw designs. Further investigation into the influence of bone density on pullout strength should be conducted in animal models. Fourth, the measurement of pullout strength at the screw/bone interface did not take into account the cortical shell of spinal vertebrae, which might have an impact on the interfacial bonding strength. However, we believe that the results regarding pullout strength provide a preliminary platform for the comparison of the postoperative stability of pedicle screws with various slit designs in spinal fusion surgery. Fifth, the volume of injected cement tested was constant (3 ml). The amount of injected cement might be an important factor determining screw holding power, and the effects of the amount of injected cement on the bone/screw interfacial strength should be studied in the future. Finally, only static loading (pullout tests on the synthetic bone) was used; other types of physiological loading were not considered. In real-life situations, the screw/bone interface is subjected to dynamic multidirectional loading. Although our loading mode did not necessarily represent actual physiological loading conditions, all of the specimens were prepared and tested in a uniform and reproducible manner, and we believe that this study provides information that could be useful for orthopedic surgeons that perform spinal fusion surgery. Further investigation into the effects of other loading methods, such as dynamic fatigue testing, might be necessary in the future.

## Conclusions

We conclude that pedicle screws combined with cement augmentation may greatly increase screw fixation regardless of screws with or without expansion. An increase in both the number of slits and the extent of screw expansion had little impact on the screw-anchoring strength. Cement augmentation is the most influential factor for improving screw pullout strength for expansive pedicle screws.

## References

[1] Riaz-ur-Rehman A, Azmatullah F, Azam F, Mushtaq M, Shah M. Treatment of traumatic unstable thoracolumbar junction fractures with transpedicular screw fixation. J Pak Med Assoc. 2011;61: 1005–1008. 22356037

[2] Fisher C, Singh S, Boyd M, Kingwell S, Kwon B, LI MJ, et al Clinical and radiographic outcomes of pedicle screw fixation for upper thoracic spine (T1-5) fractures: a retrospective cohort study of 27 cases. J Neurosurg. Spine 2009;10: 207–213. 10.3171/2008.12.SPINE0844 19320579

[3] Haq MI, Khan SA, Aurangzeb A, Ahmed E, Bhatti SN, Norman A. Radiological outcome of transpedicular screws fixation in the management of thoracolumbar spine injury. J Ayub Med Coll. Abbottabad 2015;27: 171–173. 26182768

[4] Cincu R, Lorente F de A, Gomez J, Eiras J, Agrawal A. A 10-year follow-up of transpedicular screw fixation and intervertebral autogenous posterior iliac crest bone graft or intervertebral B-Twin system in failed back surgery syndrome. Asian J Neurosurg. 2015;10: 75–82. 10.4103/1793-5482.145120 25972934 PMC4421972

[5] Koktekir E, Toktas ZO, Seker A, Akakin A, Konya D, Kilic T. Anterior transpedicular screw fixation of cervical spine: is it safe? Morphological feasibility, technical properties, and accuracy of manual insertion. J Neurosurg Spine 2015;22: 596–604. 10.3171/2014.10.SPINE14669 25815805

[6] Wu JC, Huang WC, Tsai HW, Ko CC, Wu CL, Tu TH, et al Pedicle screw loosening in dynamic stabilization: incidence, risk, and outcome in 126 patients. Neurosurg. Focus 2011;31: E9. 10.3171/2011.7.FOCUS11125 21961872

[7] Galbusera F, Volkheimer D, Reitmaier S, Berger-Roscher N, Kienle A, Wilke HJ. Pedicle screw loosening: a clinically relevant complication? Eur. Spine J. 2015;24: 1005–1016. 10.1007/s00586-015-3768-6 25616349

[8] Ko CC, Tsai HW, Huang WC, Wu JC, Chen YC, Shih YH, et al Screw loosening in the Dynesys stabilization system: radiographic evidence and effect on outcomes. Neurosurg. Focus 2010;28: E10. 10.3171/2010.3.FOCUS1052 20568916

[9] Halvorson TL, Kelley LA, Thomas KA, Whitecloud TS III, Cook SD. Effects of bone mineral density on pedicle screw fixation. Spine 1994;19: 2415–2420. 7846594 10.1097/00007632-199411000-00008

[10] Burval DJ, McLain RF, Milks R, Inceoglu S. Primary pedicle screw augmentation in osteoporotic lumbar vertebrae: biomechanical analysis of pedicle fixation strength. Spine 2007;32: 1077–1083. 17471088 10.1097/01.brs.0000261566.38422.40

[11] Hadjipavlou AG., Nicodemus CL, al-Hamdan FA, Simmons JW, Pope MH. Correlation of bone equivalent mineral density to pull-out resistance of triangulated pedicle screw construct. J Spinal Disord. 1997;10: 12–19. 9041491

[12] Chen LH, Tai CL, Lee DM, Lai PL, Lee YC, Niu CC, et al Pullout strength of pedicle screws with cement augmentation in severe osteoporosis: a comparative study between cannulated screws with cement injection and solid screws with cement pre-filling. BMC Musculoskelet. Disord. 2011;12: 33 10.1186/1471-2474-12-33 21284883 PMC3224375

[13] Chen LH, Tai CL, Lai PL, Lee DM, Tsai TT, Fu TS, et al Pullout strength for cannulated pedicle screws with bone cement augmentation in severely osteoporotic bone: influences of radial hole and pilot hole tapping. Clin Biomech. 2009;24: 613–618. 10.1016/j.clinbiomech.2009.05.002 19481845

[14] Charles YP, Pelletier H, Hydier P, Schuller S, Garnon J, Sauleau EA, et al Pullout characteristics of percutaneous pedicle screws with different cement augmentation methods in elderly spines: an in vitro biomechanical study. Orthop Traumatol Surg Res. 2015;101: 369–374. 10.1016/j.otsr.2015.01.005 25755067

[15] Renner SM, Lim TH, Kim WJ, Katolik L, An HS, Andersson GB. Augmentation of pedicle screw fixation strength using an injectable calcium phosphate cement as a function of injection timing and method. Spine 2004;29: E212–E216. 15167670 10.1097/00007632-200406010-00020

[16] Bianco RJ, Arnoux PJ, Wagnac E, Mac-Thiong JM, Aubin CE. Minimizing pedicle screw pullout risks: A detailed biomechanical analysis of screw design and placement. J. Spinal Disord Tech. 2014;19:3415–2420. 10.1097/BSD.0000000000000151 28323704

[17] Shea TM, Laun J, Gonzalez-Blohm SA, Doulgeris JJ, Lee WE 3rd, Aghayev K, et al Designs and techniques that improve the pullout strength of pedicle screws in osteoporotic vertebrae: current status. BioMed Res Int. 2014, 748393 (2014). 10.1155/2014/748393 [Review]. 24724097 PMC3958762

[18] Krenn MH, Piotrowski WP, Penzkofer R, Augat P. Influence of thread design on pedicle screw fixation. Laboratory investigation. J Neurosurg Spine 2008;9: 90–95. 10.3171/SPI/2008/9/7/090 18590418

[19] Ponnusamy KE, Iyer S, Gupta G, Khanna AJ. Instrumentation of the osteoporotic spine: biomechanical and clinical considerations. Spine J. 2011;11: 54–63. 10.1016/j.spinee.2010.09.024 21168099

[20] Bullmann V, Liljenqvist UR, Rödl R, Schulte TL. [Pedicle screw augmentation from a biomechanical perspective]. Orthopade 2010;39: 673–678. 10.1007/s00132-010-1602-8 20523969

[21] Chang MC, Liu CL, Chen TH. Polymethylmethacrylate augmentation of pedicle screw for osteoporotic spinal surgery: a novel technique. Spine 2008;33: E317–E324. 10.1097/BRS.0b013e31816f6c73 18449032

[22] Kiner DW, Wybo CD, Sterba W, Yeni YN, Bartol SW, Vaidya R. Biomechanical analysis of different techniques in revision spinal instrumentation: larger diameter screws versus cement augmentation. Spine 2008;33: 2618–2622. 10.1097/BRS.0b013e3181882cac 19011543

[23] Moon BJ, Cho BY, Choi EY, Zhang HY. Polymethylmethacrylate-augmented screw fixation for stabilization of the osteoporotic spine: a three-year follow-up of 37 patients. J Korean Neurosurg Soc. 2009;46: 305–311. 10.3340/jkns.2009.46.4.305 19893717 PMC2773385

[24] Zhuang XM, Yu BS, Zheng ZM, Zhang JF, Lu WW. Effect of the degree of osteoporosis on the biomechanical anchoring strength of the sacral pedicle screws: an in vitro comparison between unaugmented bicortical screws and polymethylmethacrylate augmented unicortical screws. Spine 2010;35: E925–E931. 10.1097/BRS.0b013e3181c5fb21 20098349

[25] Masaki T, Sasao Y, Miura T, Torii Y, Kojima A, Aoki H, et al An experimental study on initial fixation strength in transpedicular screwing augmented with calcium phosphate cement. Spine 2009;34: E724–E728. 10.1097/BRS.0b013e3181adc0e9 19752691

[26] Hashemi A, Bednar D, Ziada S. Pullout strength of pedicle screws augmented with particulate calcium phosphate: an experimental study. Spine J. 2009;9: 404–410. 10.1016/j.spinee.2008.07.001 18790679

[27] Wu XT, Jiang XJ, Zhang SD, Yang HL. Biomechanical evaluation of vertebroplasty using calcium sulfate cement for thoracolumbar burst fractures. Chin J Traumatol. 2007;10: 327–333. 18045512

[28] Yi X, Wang Y, Lu H, Li C, Zhu T. Augmentation of pedicle screw fixation strength using an injectable calcium sulfate cement: an in vivo study. Spine 2008;33: 2503–2509. 10.1097/BRS.0b013e318184e750 18978590

[29] McKoy BE, An YH. An injectable cementing screw for fixation in osteoporotic bone. J Biomed Mater Res. 2000;53: 216–220. 10813760 10.1002/(sici)1097-4636(2000)53:3<216::aid-jbm5>3.0.co;2-o

[30] Cook SD, Salkeld SL, Stanley T, Faciane A, Miller SD. Biomechanical study of pedicle screw fixation in severely osteoporotic bone. Spine J. 2004;4: 402–408. 15246300 10.1016/j.spinee.2003.11.010

[31] Gao M, Lei W, Wu Z, Liu D, Shi L. Biomechanical evaluation of fixation strength of conventional and expansive pedicle screws with or without calcium based cement augmentation. Clin. Biomech. 2011;26: 238–244. 10.1016/j.clinbiomech.2010.10.008 21084138

[32] Liu D, Zhang Y, Zhang B, Xie QY, Wang CR, Liu JB, et al Comparison of expansive pedicle screw and polymethylmethacrylate-augmented pedicle screw in osteoporotic sheep lumbar vertebrae: biomechanical and interfacial evaluations. PLoS One 2013;8: e74827 10.1371/journal.pone.0074827 eCollection 2013. 24086381 PMC3781142

[33] Gibson L, Ashby M. Cancellous Bone in Cellular Solids: Structure & Properties. 316–331 (Pergamon Press, 1988).

[34] ASTM F-543: Standard Specification and Test Methods for Metallic Medical Bone Screws, (2007).

[35] Cook SD, Salkeld SL, Whitecloud TS III, Barbera J. Biomechanical evaluation and preliminary clinical experience with an expansive pedicle screw design. J Spinal Disord 2000;13: 230–236. 10872761 10.1097/00002517-200006000-00006

[36] Choma TJ, Frevert WF, Carson WL, Waters NP, Pfeiffer FM. Biomechanical analysis of pedicle screws in osteoporotic bone with bioactive cement augmentation using simulated in vivo multicomponent loading. Spine 2011;36: 454–462. 10.1097/BRS.0b013e3181d449ec 20881517

